# Supplementary material for: ABC Transporters and CYP3A4 Mediate Drug Interactions between Enrofloxacin and Salinomycin Leading to Increased Risk of Drug Residues and Resistance
Source: Antibiotics (Basel). 2023 Feb 17;12(2):403. doi: 10.3390/antibiotics12020403 (PMC9952136; doi:10.3390/antibiotics12020403)
Supplement: Supplementary file 1 [file antibiotics-12-00403-s001.zip › antibiotics-2100561-supplementary.pdf]

Supplementary Materials

1. Primer information for CYP3A4, ABCB1/P-gp and ABCG2/BCRP

Table S1. Real-time PCR primers for Caco-2 cells.

Gene	Forward primer (F)	Reverse primer (R)
P-gp (NM_001348945.2)	CTAATGGCTTTCCTTCTGATGC	TAAGCTGATAGACGTCAGACAC
BCRP (NM_004827.3)	GCAGCAGGTCAGAGTGTGGTTTC	ACTGAAGCCATGACAGCCAAGATG
GAPDH (NM_002046.7)	CATGTTGCAACCGGGAAGGA	CAGGAGCGCAGGGTTAGTC

Table S2. Real-time PCR primers for broiler liver and small intestine.

Gene	Forward primer (F)	Reverse primer (R)
CYP3A4 (NM_001329508.2)	GTGGACTTCCTGCAGCTGAT	CCTTCTCCCTGGCAGACTTG
P-gp (NM_204894.2)	GCTGACTGTGTAGGGACTCA	GGTCCAGTTGGCCTGCAAAT
BCRP (NM_001328490.2)	CCGCTTGTCCACCAGTTACTTCAG	TTGCCATGTTAGTAGGTGCGATTCC
GAPDH (NM_204305.2)	GCAACCGTGTTGTGGACTTG	CTCCAACAAAGGGTCCTGCT

2. Methodological results of probe drugs and its products in chicken microsome

Table S3. The MRM parameters of probe drugs and its metabolites.

Compounds	Ionic mode	Parent ion (m/z)	Product ion (m/z)	De-cluster voltage (DP/V)	Collision energy (CE/V)
DM	+	272.0	215.0	31	39
DP	+	258.1	199.1	31	53
MP	+	219.1	134.0	50	20
4-OH MP	+	235.2	150.1	21	25
DCF	-	294.0	250.0	-20	-14
4-OH DCF	+	312.1	230.1	31	29
CLZ	-	168.0	132.0	-150	-22
6-OH CLZ	-	183.9	119.8	-20	-18

Table S4. Standard curve of probe substrates and products.

Compounds	Linear range	Standard curve equations	Correlation coefficient
DM	0.1-20 μg/L	y = 139158x + 306428	R = 0.9899
DP	0.2-40 μg/L	y= 1.0426x - 29795	R = 0.9999
MP	0.2-40 μg/L	y = 61882x+36485	R = 0.9997
4-OH MP	0.2-40 μg/L	y= 1.0426x - 29795	R = 0.9996
DCF	1-200 μg/L	y = 609.21x + 12324	R = 0.9963
4-OH DCF	0.2-40 μg/L	y = 54545x + 16985	R = 0.9999
CLZ	1-200 μg/L	y = 25418x + 662591	R = 0.9931
4-OH CLZ	1-200 μg/L	y = 2247.9x + 46135	R = 0.9934
HCO	0.005-1 μmol/L	y = 7e^{7}x+2382.4	R = 0.9998
TS	0.2-50 mg/L	y = 18751x+63.428	R = 0.9998

Table S5. Accuracy and precision of probe substrates and products in chicken liver microsomes.

Compounds	Concentration	Intraday RSD (%)	Interday RSD (%)	Recovery (%)
	0.2 μg/L	2.11	3.70	110.26 ± 6.80
DM	2 μg/L	2.92	1.83	104.55 ± 2.68
	20 μg/L	5.03	2.14	101.90 ± 1.00
	0.1 μg/L	0.98	5.34	104.26 ± 7.26
DP	1 μg/L	1.00	4.01	103.83 ± 1.68
	10 μg/L	8.61	1.74	100.38 ± 2.27
	0.2 μg/L	4.68	10.96	114.64 ± 6.19
MP	2 μg/L	3.30	4.76	117.09 ± 1.25
	20 μg/L	1.06	1.49	105.51 ± 1.47
	0.2 μg/L	1.94	3.80	106.72 ± 8.10
4-OH MP	2 μg/L	2.98	0.70	105.11 ± 3.88
	20 μg/L	2.13	2.64	99.80 ± 3.72
	1 μg/L	8.61	1.74	106.34 ± 9.80
DCF	10 μg/L	0.63	1.54	100.81 ± 2.01
	100 μg/L	0.61	1.97	98.07 ± 1.79
	0.2 μg/L	4.19	11.64	109.15 ± 8.57
4-OH DCF	2 μg/L	1.50	0.58	99.24 ± 2.58
	20 μg/L	1.08	0.83	98.92 ± 1.62
	1 μg/L	3.40	1.94	107.83 ± 4.56
CLZ	10 μg/L	1.89	2.67	96.25 ± 2.48
	100 μg/L	1.20	2.32	97.15 ± 1.86
	1 μg/L	7.83	5.24	98.61 ± 8.06
6-OH CLZ	10 μg/L	2.06	4.49	97.96 ± 2.49
	100 μg/L	2.20	1.32	102.34 ± 2.54
	0.01 μmol/L	1.34	1.89	97.0 ± 0.94
HCO	0.1 μmol/L	1.39	2.16	92.4 ± 0.14
	1 μmol/L	0.36	1.92	92.3 ± 0.17
	0.2 mg/L	2.73	7.42	99.74 ±1.00
TS	1 mg/L	0.95	4.58	96.93 ±1.57
	5 mg/L	0.36	2.31	100.49 ± 0.24

Table S6. LOD and LOQ of probe substrates and products.

Compounds	LOD	LOQ
DM	0.2 μg/L	0.2 μg/L
DP	0.1 μg/L	0.1 μg/L
MP	0.2 μg/L	0.2 μg/L
4-OH MP	0.2 μg/L	0.2 μg/L
DCF	1.0 μg/L	1 μg/L
4-OH DCF	0.2 μg/L	0.2 μg/L
CLZ	1.0 μg/L	1 μg/L
6-OH CLZ	1.0 μg/L	1 μg/L
HCO	0.005 μmol/L	0.005 μmol/L
TS	0.1 mg/L	0.2 mg/L

3. Methodological results of ENR, CIP and SAL in plasma

Table S7. Standard curve of ENR, CIP and SAL.

Compounds	Linear range	Standard curve equations	Correlation coefficient
ENR	20-5000 μg/L	y = 22163x-72772	R = 0.9998
CIP	20-5000 μg/L	y = 14448x-588385	R = 0.9997
SAL	0.2-50 μg/L	y = 24030x+9118.9	R = 0.9999

Table S8. LOD and LOQ of ENR, CIP and SAL.

Compounds	LOD	LOQ
ENR	10 μg/L	20 μg/L
CIP	10 μg/L	20 μg/L
SAL	0.2 μg/L	0.2 μg/L

Table S9. Accuracy and precision of ENR, CIP and SAL.

Compounds	Concentration	Intraday RSD (%)	Interday RSD (%)	Recovery (%)
	20 μg/L	1.19	4.80	95.70 ± 1.40
ENR	100 μg/L	1.47	6.85	99.85 ± 2.43
	1000 μg/L	2.93	1.02	107.02 ± 1.66
	20 μg/L	6.37	6.29	106.95 ± 3.74
CIP	100 μg/L	1.66	2.15	96.24 ± 3.20
	1000 μg/L	2.94	1.16	105.90 ± 1.61
	0.2 μg/L	5.67	12.83	105.05 ± 5.89
SAL	5 μg/L	11.02	14.72	102.58 ± 4.54
	50 μg/L	10.08	14.49	96.19 ± 5.22

4. Pharmacokinetic parameters of ENR, CIP and SAL

Table S10. Main pharmacokinetic parameters of ENR in broilers after single or combined administration (n=6, Mean ± SD).

Parameter (Unit)	ENR (7.5 mg/kg)	SAL+ ENR	SAL (5 d) + ENR
Ke (1/h)	0.07 ± 0.01	0.09 ± 0.01	0.09 ± 0.01
AUC_{0-36h} (h*mg/L)	12.75 ± 3.99	16.73 ± 1.29*	15.32 ± 1.29*
$AUC_{0-\infty}$ (h*mg/L)	13.50 ± 4.08	17.47 ± 1.45*	16.02 ± 1.45*
MRT_{0-36h} (h)	8.29 ± 1.03	9.06 ± 0.52	8.35 ± 0.52
$MRT_{0-\infty}$ (h)	10.75 ± 1.76	10.64 ± 0.90	10.05 ± 0.90
C_{max} (mg/L)	1.49 ± 0.54	1.48 ± 0.16	1.60 ± 0.16
T_{max} (h)	1.50 ± 0.55	2.67 ± 1.03	2.17 ± 1.03
$t_{1/2}$ (h)	7.45 ± 1.71	8.65 ± 0.54	8.13 ± 0.86

Note: Ke, terminal elimination rate; AUC, area under the curve; C_{max}, maximum plasma concentration; MRT, mean residence time; $T_{1/2}$, elimination half-life; T_{max}, time to reach the C_{max}. *statistical significance compared with ENR (7.5 mg/kg) is $P < 0.05$.

Table S11. Pharmacokinetic parameters of CIP in chickens after single or combined administration (n=6, Mean ± SD).

Parameter (Unit)	ENR (7.5 mg/kg)	SAL + ENR	SAL (5 d) + ENR
Ke (1/h)	0.16 ± 0.02	0.06 ± 0.03***	0.07 ± 0.02***
AUC_{0-24h} (h*mg/L)	0.54 ± 0.18	1.29 ± 0.15***	1.22 ± 0.16***
AUC0-∞ (h*mg/L)	0.67 ± 0.21	1.84 ± 0.32***	1.57 ± 0.13***
MRT0-24h (h)	4.90 ± 0.36	9.35 ± 1.06***	9.11 ± 0.45***
MRT0-∞ (h)	7.56 ± 1.10	14.11 ± 3.18**	15.79 ± 3.83***
Cmax (mg/L)	0.08 ± 0.03	0.10 ± 0.03	0.11 ± 0.02
Tmax (h)	3.33 ± 1.03	3.20 ± 1.10	3.00 ± 1.10
t1/2 (h)	4.51 ± 0.60	11.12 ± 4.77**	9.96 ± 2.40*

Note: Ke, terminal elimination rate; AUC, area under the curve; C_{max}, maximum plasma concentration; MRT, mean residence time; $T_{1/2}$, elimination half-life; T_{max}, time to reach the C_{max}. *Statistical significance compared with ENR group (7.5 mg/kg) is $P < 0.05$; **Statistical significance compared with ENR group (7.5 mg/kg) is $P < 0.01$. ***Statistical significance compared with ENR group (7.5 mg/kg) is $P < 0.001$.

Table S12. Pharmacokinetic parameters of SAL in chickens after single or combined administration (n=6, Mean ± SD)

Parameter (Unit)	SAL (6 mg/kg)	SAL+ ENR	ENR (5 d) + SAL
Ke (1/h)	0.05 ± 0.01	0.10 ± 0.06	0.04 ± 0.02
AUC_{0-48h} (h*μg/L)	218.96 ± 42.32	148.30 ± 32.07*	409.09 ± 39.12***
$AUC_{0-\infty}$ (h*μg/L)	264.78 ± 40.60	153.25 ± 31.01*	495.16 ± 97.71***
MRT_{0-48h} (h)	5.53 ± 1.16	6.30 ± 1.32	12.05 ± 1.84***
$MRT_{0-\infty}$ (h)	7.53 ± 2.39	8.68 ± 3.70	18.15 ± 5.35***
C_{max} (μg/L)	82.02 ± 29.08	54.76 ± 18.55	134.32 ± 48.37*
T_{max} (h)	0.42 ± 0.30	0.29 ± 0.10	0.29 ± 0.10
$t_{1/2}$ (h)	14.17 ± 3.10	14.00 ± 12.27	16.46 ± 6.53

Note: Ke, terminal elimination rate; AUC, area under the curve; C_{max}, maximum plasma concentration; MRT, mean residence time; $T_{1/2}$, elimination half-life; T_{max}, time to reach the C_{max}. *statistical significance compared with SAL group (6.0 mg/kg) is P < 0.05; **statistical significance compared with SAL group (6.0 mg/kg) is P < 0.01; ***statistical significance compared with SAL group (6.0 mg/kg) is P < 0.001.

Table S13. Molecule docking simulation of the compound with CYP3A4.

Compounds	Hydrogen bonding site with CYP3A4 (PDB ID: 6MA7)	Score
ENR	A/ARG:105; **A/Glu: 381**; A/ARG:382; A/ARG:447	6.92
SAL	A/Lys: 212; **A/Glu: 381**	10.29

Table S14. Molecule docking simulation of the compound with P-gp.

Compounds	Hydrogen bonding site with P-gp (PDB ID: 6C0V)	Score
ATP	A/ASP177, **A/LYS181**, **A/GLU243**, A/ALA823, **A/LYS826**	9.1007
ENR	**A/LYS181**, **A/GLU243**, **A/LYS826**, A/SER831	5.6517
SAL	**A/LYS181**, 2 A/ASN296, A/GLU353, A/SER831	8.4186

Table S15. Molecule docking simulation of the compound with BCRP.

Compounds	Hydrogen bonding site with P-gp (PDB ID: 6C0V)	Score
ATP	A/ARG123, **A/LYS124**, A/TRY150, A/GLN153, A/ASP154, **A/ASP237**, A/LYS490, A/CXL701	7.4629
ENR	A/SER114, **A/ ASP237**, A/ARG698	5.6517
SAL	A/SER115, **A/LYS124**, A/GLU569, A/CXL701	8.4186

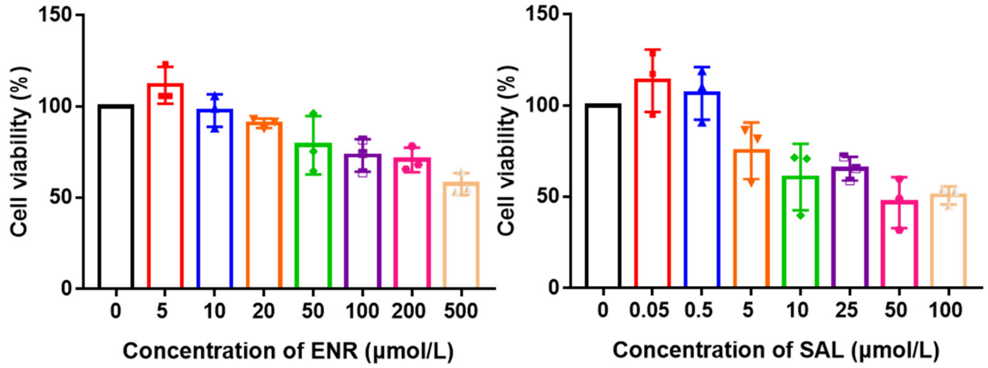

Figure S1. Effects of ENR and SAL exposure on the viability of Caco-2 cells.

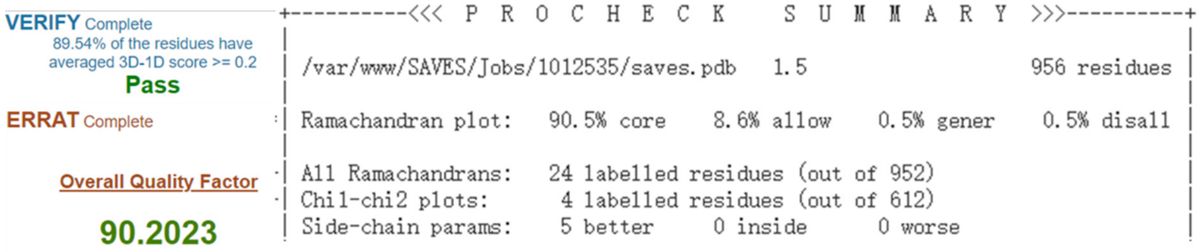

Figure S2. The evaluation results of the CYP3A4 (PDB ID: 6MA7) model with VERIFY3D, ERRAT and PROCHECK software.

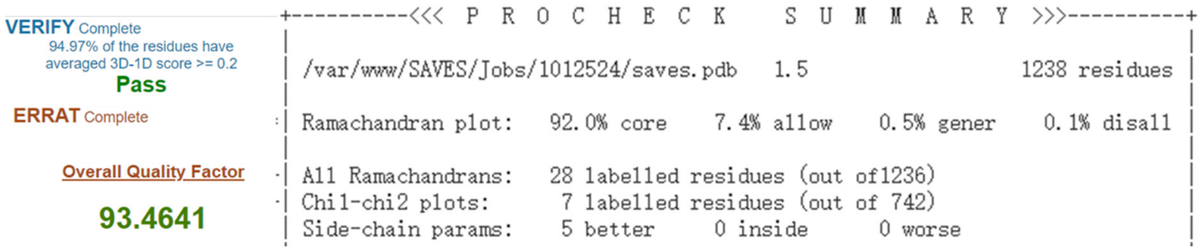

Figure S3. The evaluation results of the P-gp (PDB ID: 6C0V) model with VERIFY3D, ERRAT and PROCHECK software.

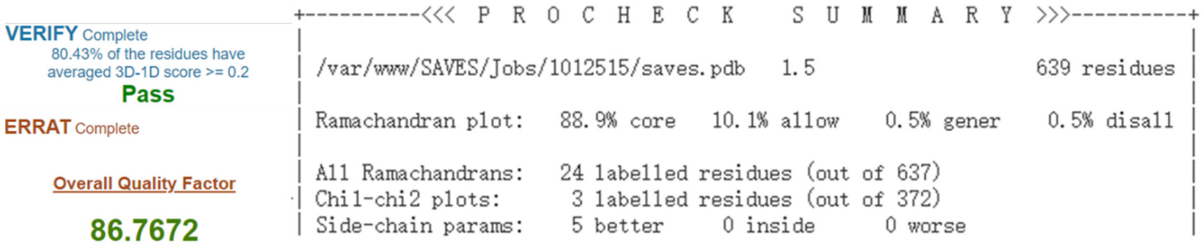

Figure S4. The evaluation results of the BCRP (PDB ID: 6ETI) model with VERIFY3D, ERRAT and PROCHECK software.
